# 

**Published:** 1898-12-31

**Authors:** 


**Notices of Births, Deaths, and Marriages** are inserted in this column at a charge of 2s. 6d. for an announcement not exceeding four lines.

### NOTICE TO CORRESPONDENTS.

All MS., Letters, Books for Review, and other matters intended for the Editor should be addressed The Editor, "The Hospital," The Hospital Building, 28 & 29, Southampton Street, Strand, W.C.

The Editor cannot undertake to return rejected MS., even when accompanied by stamped directed envelope.

**Notice to Advertisers and Subscribers.**

All Advertisements, Orders for Copies of The Hospital, and Business Communications should be addressed to **THE MANAGER (Not to the Editor),** The Hospital, The Hospital Building, 28 & 29, Southampton Street, Strand, London, W.C.

The Cost of Subscription, post free, is as follows:—Per week, 2½d.; per quarter, 2s. 8d.; per half-year, 5s. 3d.; per annum, 10s. 6d. Foreign:—Per annum, 15s. 2d., payable, in all cases, in advance.

**NOTICE.—Vol. XXIV. of "The Hospital" (April, 1898, to September, 1898), in handsome cloth binding, is now on sale at the Office of this Journal, price 7s. 6d. Cases for binding the Numbers contained in this Volume 1s. 6d. Index to Vol. XXIV., 2½d., post free.**

**The Monthly Part for January will be ready on 2nd January. Price 6d., by post 8d.**

### BOOKS RECEIVED.

Her Majesty's Stationery Office.

"Supplement to the Twenty-seventh Annual Report of the Local Government Board, containing the Report of the Medical Officer."

Mayne and Boyd, Belfast.

"A Consumptive Sanatorium for the People." By Dr. Nahm. Translated and published with an introduction by William Caldwell, M.A., M.D.

Sampson Low, Marston, and Co.

"Twentieth Century Practice of Medicine." Edited by Thomas L. Stedman, M.D. Volume XV.

"Manual of First Aid." By J. A. Austin, M.D.

Harald Bruhn, Braunschweig.

"Jahresbericht über die Fortschritte in der Lehre von den Pathogenen Mikro-organismen." By Dr. Med. P. von Bauengarten and Dr. Med. F. Tangl. Twelfth year. 1896.

Charles William Deacon and Co.

"Before Good Night: A Little Story Told to a Little Child."

Universal Cookery and Food Association.

"Simple Cookery for the People."

Henry Froude.

"The Micro-organism of Faulty Rum." By V. H. Veley, M.A., F.R.S., and Lilian J. Veley (née Gould).

Sanders' Printing Office, Washington.

"The Difference Between the Two Systems of Teaching Deaf Mute Children."

W. B. Saunders, Philadelphia.

"Nursing: Its Principles and Practice." By Isabel Adams Hampton.

J. and A. Churchill.

"Vitality: An Appeal, an Apology, and a Challenge." By Lionel S. Beale.

Henry J. Glaisher.

"On So called Spasmodic Asthma." By Ernest Kingscote, M.B., C.M.

The Chemist and Druggist.

"The Chemists and Druggists' Diary, 1899."

The Scientific Press.

"Hospital Expenditure: The Commissariat." [Reprinted from The Hospital.]

### Scraps and Gleanings.

It is hoped that the Cottage Hospital at Bishop Auckland will be ready for occupation next spring.

In the Hahnemann Hospital, New York, a hospital bed cot has been endowed for the perpetual use of fractured footballers, the number of accidents resulting from the game, as played in the United States, being so numerous.

It is estimated that a sum of £2,000 is required to enable the Belfast Maternity Hospital to be rebuilt. At a meeting held for the purpose of inaugurating this fund a subscription of £1,000 was promised, provided the sum mentioned should be first raised.

The cost of erection of the Victoria Cottage Hospital at Woking will be £3,100. It is to contain accommodation for 18 patients.

The new Mater Hospital at Belfast is rapidly approaching completion. An "envelope collection" in aid of the funds for the furnishing of the children's ward has been organised amongst the school children of the diocese and also outside.

An appeal is being made in the local Press for the establishment of a cottage hospital in Ventnor, which it is stated is a long-felt want, the nearest institution to which cases of accident requiring more than temporary help can go being Ryde Infirmary.

### The Student of Medicine.

### APPOINTMENTS.

P. S. Blaker, M.R.C.S., L.R.C.P.Lond., appointed House Physician at the City of London Hospital for Diseases of the Chest, Victoria Park. G. Booth, M.D.Durh., appointed Honorary Surgeon to the Chesterfield and North Derbyshire Hospital. R. Burgess, M.B., C.M., appointed Certifying Factory Surgeon for the Stanley District. F. Edmunds, M.R.C.S.Eng., L.R.C.P.-Lond., appointed Honorary Surgeon to the Chesterfield and North Derbyshire Hospital. Dr. J. Forster, appointed Medical Officer for the Conisbrough District of the Doncaster Union. W. A. Fuller, M.R.C.S., L.R.C.P., appointed Assistant House Surgeon to the Cardiff Infirmary. J. F. Hall, M.B., M.Ch., appointed Assistant Physician to the Hospital of St. Francis, New Kent Road, S.E. P. Kitchin, L.S.A., appointed Medical Officer for the Box and Ditteridge District of the Chippenham Union. G. P. Matthew, M.A., M.D., B.C.Cantab., M.R.C.S., L.R.C.P., appointed Assistant Surgeon to the Provincial Hospital, Port Elizabeth. C. H. Melland, M.B.Lond., M.R.C.S., appointed Resident Medical Officer to the Manchester Royal Infirmary. E. Pratt, M.B.Lond., F.R.C.S., appointed Resident Medical Officer to the Cardiff Infirmary. P. W. Rattray, M.B.-Aberd., appointed Medical Officer to the Highgate Hill Workhouse of the Parish of St. Mary, Islington. W. Rawes, M.D., F.R.C.S., appointed Medical Superintendent of St. Luke's Hospital, London, E.C. W. Robertson, M.D.Glasg., D.P.H.-Edin. and Glasg., appointed Medical Officer of Health and Police Surgeon to the City of Perth. B. Stevenson, L.D.S., appointed Clinical Dental Assistant to the Hospital of St. Francis, New Kent Road, S.E. H. A. Stonham, L.R.C.P., M.R.C.S., D.P.H., appointed Public Vaccinator for the Stepney Union. J. A. Turner, M.B., D.P.H., appointed County Medical Officer of Health for Hertfordshire.

### PASS LISTS.

#### University of London.

#### M.D. EXAMINATION.—1898.

Medicine.—P. E. Adams, St. Bartholomew's Hospital; A. P. Allan, B.S., Guy's Hospital; J. H. Bodman, B.S., University College, Bristol, and St. Bartholomew's Hospital; W. F. V. Bonney, B.S., Middlesex Hospital; *J. A. O. Briggs, St. Bartholomew's Hospital; Maud M. Chadburn, London School of Medicine and Royal Free Hospital; F. N. Cookson, Middlesex Hospital; M. Dixon, B.Sc., University College; R. W. Dodgson (gold medal), St. Mary's Hospital; E. G. D. Drury, B.S., St. Bartholomew's Hospital; G. R. Elwin, St. Mary's Hospital; A. H. Evans, B.S., Westminster Hospital; J. W. Haines, B.S., St. Bartholomew's Hospital; A. Heath, St. Bartholomew's Hospital; A. Howell, St. Mary's Hospital; T. H. Hunt, B.S., Owens and Yorkshire Colleges; *J. Hussey, St. Bartholomew's Hospital; W. H. Jewell, B.S., Guy's Hospital; J. L. Jones, B.S., University College; C. H. J. Lockyer, B.S., Charing Cross Hospital; A. A. Martin, B.S., St. Mary's Hospital; Elizabeth J. Moffett, B.Sc., London School of Medicine and Royal Free Hospital; E. Pratt, St. Bartholomew's Hospital; G. B. Price, B.S., University College, Bristol, and St. Bartholomew's Hospital; W. T. G. Pugh, B.S., Middlesex Hospital; A. W. Sanders, St. Mary's Hospital; W. G. Savage, B.Sc., University College; H. J. Scharlieb, B.S., University College; A. W. Sikes, B.S., B.Sc., St. Thomas's Hospital; E. I. Sprigg's, Guy's Hospital; W. H. B. Stoddart, B.S., University College; F. H. Thiele, B.Sc., University College; E. Thomas, B.S., University College; E. J. Toye, B.S., B.Sc., St. Bartholomew's Hospital; Ethel M. Vaughan, B.S., London School of Medicine and Royal Free Hospital; W. B. Warde, St. Bartholomew's Hospital; T. H. Wells, Middlesex Hospital. * Obtained the number of marks qualifying for the gold medal.

State Medicine.—W. E. Dixon, B.S., B.Sc., St. Thomas's Hospital; W. J. Potts, M.D., Owens and University Colleges.

#### M.S. EXAMINATION.

C. H. Fagge, Guy's Hospital; C. E. M. Kelly, M.D., Owens College; J. S. Sloane, B.Sc., St. Bartholomew's Hospital; W. Turner, King's College.

